# Sleep Quality, Empathy, and Mood During the Isolation Period of the COVID-19 Pandemic in the Canadian Population: Females and Women Suffered the Most

**DOI:** 10.3389/fgwh.2020.585938

**Published:** 2020-10-23

**Authors:** Veronica Guadagni, Alberto Umilta’, Giuseppe Iaria

**Affiliations:** ^1^Department of Physiology and Pharmacology, Cumming School of Medicine, University of Calgary, Calgary, AB, Canada; ^2^Hotchkiss Brain Institute, Cumming School of Medicine, University of Calgary, Calgary, AB, Canada; ^3^O'Brien Institute for Public Health, Cumming School of Medicine, University of Calgary, Calgary, AB, Canada; ^4^NeuroLab, Department of Psychology, Faculty of Arts, University of Calgary, Calgary, AB, Canada; ^5^Alberta Children's Hospital Research Institute, University of Calgary, Calgary, AB, Canada

**Keywords:** coronavirus, insomnia, emotions, depression, anxiety

## Abstract

**Objective:** To investigate the sex and gender differences in the impact of the isolation period implemented in response to the COVID-19 pandemic on individuals' sleep quality, empathy, and mood.

**Design:** Data were collected between March 23 and June 7, 2020 on a sample of volunteers in the Canadian population. Six hundred and thirty-eight volunteers completed an online survey (~30 min).

**Main Outcome and Measures:** We first examined biological sex, gender, and sexual identity differences (both components of the ampler concept of gender) in sleep, empathy, and mood disturbances. Then, we assessed changes in sleep and mood over the course of the isolation period and tested for significant relationships between sleep variables, mood, and empathy.

**Results:** We analyzed complete data for 573 participants (112 males and 459 females, 2 undisclosed, mean ± SD age = 25.9 ± 10.5 years, mean ± SD education = 16.2 ± 2.9 years). As compared to males, female participants reported lower quality of sleep, lower sleep efficiency, and greater symptoms of insomnia, anxiety, depression, and trauma. In addition, females reported higher scores than males on the IRI empathy scale and all its subcomponents. Similar results were found when stratifying by gender. Sleep and mood disturbances increased over the course of the isolation period in the whole sample. The most significant predictors of poor quality of sleep and insomnia were depression, anxiety, and trauma scores, especially in females; higher empathy trait was associated with higher depression, anxiety, and trauma scores, perhaps indicating a more positive role of fear and anxiety responses to the pandemic crisis.

**Significance and Conclusions:** Sex and gender differences seem to play a role in the individuals' psychological and behavioral reactions to the COVID-19 pandemic. These differences need to be considered in planning targeted psychological interventions.

## Introduction

The novel severe acute respiratory syndrome coronavirus 2 (SARS-CoV-2), known as Coronavirus disease 2019 (COVID-19), appeared in the province of Wuhan, China, at the end of 2019 and quickly spread across several countries in the word (1). On March 11th 2020, the World Health Organization declared the Coronavirus outbreak a *Pandemic* (2). In absence of pharmaceutical interventions, shelter in place at home and social distancing were globally deemed as the best strategy to stop the spread of the virus (3). On January 27, 2020, the first COVID-19 case was confirmed in Canada. In mid-March, all of Canada's provinces declared states of local emergency and implemented various levels of mandatory isolation with school and daycare closures, restrictions on gatherings, closures of non-essential businesses, restrictions on entry, and mandatory quarantine for travelers. As for August 25, 2020, there have been approximately 125,645 COVID-19 confirmed cases in Canada, with 4,870 active cases and 9,083 deaths.

Although effective in containing the spread of COVID-19, isolation and social distancing caused an interruption in the normal routine of many people in the word (4), with school being closed and parents trying to balance remote working, childcare and house management (5). This has led to changes and disruption of individuals' mental well-being and sleep schedule (6), similar to those observed following previous natural disasters (7–9). To date, only a few studies have examined the changes in sleep quality and mood during the COVID-19 pandemic in both the general population and health care professionals. Casagrande et al. (10) found that 57.1% of responders to an online survey reported poor quality of sleep, 32.1% reported increased symptoms of anxiety, 41.8% increased distress, and 7.6% reported symptoms of post-traumatic stress disorder (PTSD). Sleep disorders and anxiety disorders were more prevalent in women, those unemployed, and those who were worried about being infected with COVID-19 (or knew people who died due to COVID-19). These findings are consistent with other studies conducted in the Italian (11–13), and Chinese populations (14–17), some of the most affected, confirming the significant negative impact of the pandemic on mental health. Although these studies provide a significant contribution to understanding the impact of COVID-19 on the human well-being, the effects of sex and gender in response to the pandemic, as well as the deterioration and progression of the individuals' mental health over the course of the isolation period, remain unknown.

Sex and gender differences, seem to play a role in the individuals' psychological and behavioral reactions to the pandemic (18). While often used interchangeably the two terms indicate very different things. Sex refers to a biological construct primarily associated with physical and physiological features including genes, hormones and anatomical and physiological characteristics (19). Gender refers instead to socially constructed roles, behavior, expressions, and identities (19). To date, there is no standard method to assess gender. However, recent studies have pointed out to the need to assess sexual identity (i.e., straight, gay etc.) and gender identity (man or woman), both part of the ampler definition of gender, separately from biological sex (male and female) (20). Both biological sex and gender have been shown to be associated with pattern of exposure, treatment, and behavioral changes associated with COVID-19. Biological sex seems to be associated with the infection and mortality rates, with higher numbers of men suffering greater health consequences from the virus (21, 22). These sex differences have been thought to be associated with the different immune response in the two sexes, with a different distribution of the ACE 2 receptors where the coronavirus binds, and with potential protective effects of estrogens (22). Gender, on the other hand, has been shown to play a bigger role in pattern of exposures to the virus (gender influences where people are spending time), and in the behavioral reactions to the pandemic (18).

Here, we investigated the effects of sex and gender in response to the isolation period of the pandemic, in the context of different critical elements of the individuals' mental well-being, that are sleep quality, empathy, and individual mental health status of depression, anxiety and post-traumatic stress symptoms. Of particular interest is empathy, defined as the ability to understand another individual's mental state in terms of emotions, feelings and thoughts (23), being an important aspect to consider when examining individuals' reactions to the pandemic. Empathy is in fact a fundamental process underlying the ability of caring for others and, as such, higher empathy for others may translate to higher compliance to public health rules. The concept of empathy can be further separated into cognitive and emotional components (23–27); here we focus solely on the emotional aspect of empathy. Similarly, sleep is well-known to be crucial for well-being and proper neurocognitive performance (28). Several studies have confirmed the negative impact of sleep loss on individuals' mood and emotional processing (29) including empathy (30–33). Based on this evidence, one would expect that the relationship between sleep quality and emotional processing will hold during the COVID-19 pandemic. To date, however, there is no evidence that this is the case.

It is known that sleep, empathy and mental health status may differ between the two sexes. Previous studies have in fact highlighted sex and gender differences in empathy (34), with females usually reporting higher scores as compared to males. Similarly, sleep architecture and quality differs in the two sexes with females having an overall better quality of sleep (35) but higher symptoms of insomnia (36). Males, on the other hand, tend to have more sleep disordered breathing pathologies such as obstructive sleep apnea (37). The negative effects of sleep loss on cognition also seem to be differential in the two sexes due to hormonal effects (38). Finally, mood disorders are more prevalent in females as compared to males and recent studies have tried to explain these differences highlighting how immune mechanisms may differently contribute to stress susceptibility and associated mood disorders (39). However, how these sex differences manifest during the isolation in response to the pandemic is still unclear.

In this study, we investigated if sex and gender are differently associated with sleep, empathy and mental health during the isolation that was implemented to stop the spread of COVID-19, and if the increased number of days spent in isolation heightened individuals sleep disturbances and mental health concerns. Secondarily, we investigated the most significant predictors of sleep quality during the isolation in the whole sample first, and then in subgroups stratified by sex and gender. The findings of this study may provide important insights to be considered when planning personalized psychological interventions to counterbalance the negative effects of the isolation period on sleep and mental health.

## Methods

### Participants

We recruited 638 volunteers through the University of Calgary Research Participation System and COVID-19 research page, social media and word of mouth. Collected data were anonymous and participants could voluntary withdraw from the study at any time. The final complete dataset included 573 Canadian volunteers (112 males and 459 females, 2 undisclosed, mean ± SD age = 25.9 ± 10.5 years, mean ± SD education = 16.2 ± 2.9 years). Participants' demographics and isolation status are reported in Table 1. Gender breakdown is reported in Table 2. The study was reviewed and approved by the local research ethics board (REB20-0650), and participants provided an electronic informed consent before the study began.

**Table 1:** Participants' demographics and COVID-19 status.

	**M ± SD Whole sample**	**M ± SD Males**	**M ± SD Females**
Age	25.9 ± 10.5	26.2 ± 9.4	25.9 ± 10.7
Education total	16.2 ± 2.9	16.0 ± 3.2	16.2 ± 2.9
**Ethnicity**	***N*** **(%) Whole sample**	***N*** **(%) Males**	***N*** **(%) Females**
White	321 (56%)	55 (49.1%)	266 (58%)
Afro-American	2 (0.3%)	–	2 (0.4%)
East-Asian	70 (12.2%)	18 (16.1%)	52 (11.3%)
South-Asian	101 (17.6%)	20 (17.9%)	79 (17.2%)
African	13 (2.3%)	5 (4.5%)	8 (1.7%)
Indigenous	2 (0.3%)	–	2 (0.4%)
Latino	14 (2.4%)	5 (4.5%)	9 (2.0%)
Mixed-race	32 (5.6%)	6 (5.4%)	26 (5.7%)
Other	18 (3.1%)	3 (2.7%)	15 (3.3%)
**Neuro/psychiatric condition**	***N*** **(%) Whole sample**	***N*** **(%) Males**	***N*** **(%) Females**
NO	439 (76.9)	87 (77.7%)	350 (76.3%)
YES	53 (9.2%)	9 (8.0%)	44 (9.6%)
YES (non-medicated)	53 (9.2%)	9 (8.0%)	44 (9.6%)
Concussion	27 (4.7)	6 (5.4%)	21 (4.6%)
**Current situation Days (M** **±** **SD** **=** **42.2** **±** **22.1)**	***N*** **(%) Whole sample**	***N*** **(%) Males**	***N*** **(%) Females**
Self-isolation	112 (19.5)	26 (23.2%)	85 (18.5%)
Quarantine	48 (8.4%)	11 (9.8%)	36 (7.8%)
Social distancing	406 (70.9)	72 (64.3%)	334 (72.8%)
None	7 (1.2%)	3 (2.7%)	4 (0.9%)
**Know someone with COVID (*****n*** **=** **336)**	***N*** **(%) Whole sample**	***N*** **(%) Males**	***N*** **(%) Females**
NO	247 (73.3%)	38 (33.9%)	209 (45.5%)
Myself	4 (1.2%)	1 (0.9%)	3 (0.7%)
A friend	54 (16%)	7 (6.3%)	47 (10.2%)
A relative	32 (9.5%)	3 (2.7%)	29 (6.3%)
**Know someone who died with COVID (*****n*** **=** **336)**	***N*** **(%) Whole sample**	***N*** **(%) Males**	***N*** **(%) Females**
NO	318 (94.4%)	48 (42.9%)	270 (58.8%)
A friend	11 (3.3%)	–	11 (2.4%)
A relative	8 (2.4%)	1 (0.9%)	7 (1.5%)

*M ± SD, Mean ± Standard Deviation; N, number*.

**Table 2:** Participant's gender breakdown.

		***N* (%)**
Biological sex	Male	112 (19.5%)
	Female	459 (80.1%)
	Undisclosed	2 (0.3%)
Gender ID	Woman	460 (80.6%)
	Man	105 (18.4%)
	Non-binary	5 (0.9%)
	Undisclosed	1 (0.2%)
Sexual ID	Straight	508 (88.7%)
	Gay	16 (2.8%)
	Bisexual	42 (7.3%)
	Other	7 (1.2%)

### Experimental Protocol

Participants were asked to complete an online survey (~30 min). The survey included a demographic questionnaire inquiring about age, years of formal education, ethnicity, history of neurological/psychiatric illness, medications, biological sex, gender identity, and sexual identity. The following questions were used to inquire about biological sex, gender identity and sexual identity separately: (1) what is your biological sex? Male/female; (2) what is your gender identity? Man, Woman, trans-sexual woman/man, non-binary, other; (3) what is your sexual identity? Straight, gay, bisexual, or other.

Four COVID-related questions inquired about isolation/social distancing status, length of the isolation, positivity to COVID-19, or knowledge of individuals infected or who died because of COVID-19.

The demographic questionnaire was followed by 6 questionnaires assessing sleep, mood and empathy. Self-reported sleep quality was assessed with the Pittsburgh Sleep Quality Index (PSQI) (40) and with the Insomnia Severity Index (ISI) (41), a questionnaire that assesses symptoms of insomnia. Both PSQI and ISI are validated questionnaire with Cronbach's alphas of 0.69 (42) and 0.90 (41), respectively. PSQI total score (5)≥ indicative of poor quality of sleep (40) was computed by adding responses to 7 subcomponents: (1) subjective sleep quality, (2) latency, (3) duration, (4) efficiency (hours in bed/hours sleeping), (5) sleep disturbance, (6) sleep medications, and (7) daytime dysfunction. The scores on the duration, latency and efficiency were also analyzed as separate continuous variables. Additionally, we calculated the total score for the ISI with scores ≥9 indicative of clinical insomnia (41).

Participants also completed the State-Trait Anxiety Inventory (43) which has a Cronbach's alpha for the total scores ranging from 0.86 to 0.95 (44), and the Beck Depression Inventory (BDI) (45) with a Cronbach's alpha of 0.81 for non-psychiatric population, and the Davidson Trauma Scale (46) with a Cronbach's alpha of 0.95 (47). The STAI was used to assess participants' current (state) and general (trait) anxiety symptoms, and the BDI was used to evaluate participants' depressive traits (scores >17 indicating borderline depression), while the Davidson Trauma Scale assessed trauma. Finally, the Interpersonal Reactivity Index (IRI) (48) assessed empathy with four different subscales: *Perspective Taking*, the individual's ability to take others' perspective, *Fantasy*, the ability to identify with characters of movies and books, *Empathic Concern*, the feelings of concern and compassion for others, and *Personal Distress*, the negative feeling of distress while observing someone in a negative situation. The IRI Cronbach's alpha ranges from 0.70 to 0.78 (48).

### Data Analyses

Data were analyzed using IBM SPSS Statistics for Windows, version 25.0 (IBM, Armonk, NY, USA).

We computed descriptive statistics for questionnaires' scores for the whole sample and for males and females separately. Q-Q plots were examined to assess the normal distribution of the data.

First, Kruskal-Wallis non-parametric tests were used to compare participants' questionnaires scores between biological sexes, gender identities, and sexual identities. The choice of non-parametric test was due to the large sample size difference among the subgroups. We then used linear regressions to examine the relationship between number of days spent in isolation and questionnaires' scores separately for males and females. These analyses show the progression of insomnia, depression, and trauma with increasing length of the isolation period. Finally, we ran a series of multiple linear regressions (MLRs) with PSQI total score, sleep duration, sleep latency, sleep efficiency, and ISI total score as dependent variables in separate models, age as forced confounding factor, and scores on the IRI, BDI, STAI (trait and state), and total trauma as independent predictors. We ran the MLRs analyses in the whole sample first, and then stratified by biological sex (20).

All analyses were two-tailed and statistical significance was set at *p* < 0.05. Bonferroni-Holm correction was applied to correct for multiple comparisons and to reduce experiment wise error.

## Results

### Questionnaires' Descriptive Findings

The Q-Q plots revealed that the data was normally distributed.

Three hundred and eighty-three participants (66.8%) reported poor quality of sleep, and 225 (39.2%) reported clinical insomnia. The average score on the BDI scale was 13.1 representing normal and mild mood swings. Both anxiety state and trait were heightened in the whole sample with average normative scores of 55 and 59, exceeding the cut off for clinically significant anxiety of 40 (43). Scores on the Davidson Trauma Scale were also heightened as compared to the general population with an average total trauma score of 37.9, which according to Davidson classification identifies subthreshold PTSD with impairments (46).

### Sex and Gender Differences in Sleep, Mood, and Empathy

Females compared to males reported lower quality of sleep (*p* = 0.023), sleep efficiency (*p* = 0.023), and greater symptoms of insomnia (*p* = 0.021). When correcting for multiple comparisons these differences were not significant anymore. Females also reported significantly higher symptoms of anxiety (both state and trait *p* < 0.001), depression (*p* < 0.001), and greater distress in relation to a traumatic event in both severity (*p* < 0.001) and frequency domains (*p* < 0.001). However, females reported higher scores on the IRI empathy scale (*p* < 0.001) and all its subcomponents (all *p* < 0.01). Please refer to Table 3 for complete statistics.

**Table 3:** Participants' questionnaires data: biological sex differences.

	**M ± SD whole sample**	***N***	**M ± SD Males**	***N***	**M ± SD Females**	***N***	*p*-value^*^	**Cohen *D_***z***_***
PSQI total	6.0 ± 2.7	572	5.4 ± 2.7	112	6.1 ± 2.8	458	**0.023**	0.25
PSQI latency (min)	55.9 ± 61.7	562	67.2 ± 75.1	110	53.1 ± 57.8	450	0.212	0.18
PSQI duration (hrs)	7.6 ± 1.4	572	7.6 ± 1.2	112	7.6 ± 1.4	458	0.825	0
PSQI efficiency (%)	88.7 ± 14.1	572	91.3 ± 13.5	112	88.1 ± 14.2	458	**0.021**	0.22
ISI	7.5 ± 4.8	573	6.0 ± 4.5	112	7.8 ± 4.9	459	**<0.001**	0.36
STAI state (raw)	42.9 ± 12.2	544	38.2 ± 12.2	102	44.0 ± 12.0	440	**<0.001**	0.47
STAI trait (raw)	44.1 ± 11.9	541	39.9 ± 12.3	101	45.1 ± 11.6	438	**<0.001**	0.42
BDI	13.1 ± 10.0	573	9.7 ± 9.9	112	14.0 ± 9.9	459	**<0.001**	0.43
Trauma severity	20.0 ± 13.4	552	15.1 ± 13.0	105	21.2 ± 13.2	445	**<0.001**	0.46
Trauma frequency	17.8 ± 13.2	552	12.9 ± 12.9	105	19.0 ± 13.0	445	**<0.001**	0.46
Total trauma	37.9 ± 26.1	552	28.1 ± 25.4	105	40.2 ± 25.7	445	**<0.001**	0.47
IRI total	63.8 ± 15.7	573	54.7 ± 13.4	112	65.9 ± 15.5	459	**<0.001**	0.72
IRI perspective taking	15.8 ± 5.0	573	14.6 ± 5.4	112	16.1 ± 4.8	459	**0.006**	0.27
IRI fantasy	15.3 ± 6.6	573	13.0 ± 6.1	112	15.9 ± 6.6	459	**<0.001**	0.43
IRI empathic concern	20.2 ± 4.9	573	17.3 ± 4.7	112	20.8 ± 4.8	459	**<0.001**	0.75
IRI personal distress	11.0 ± 5.3	573	8.8 ± 4.4	112	11.5 ± 5.4	459	**<0.001**	0.50

*M ± SD, Mean ± Standard Deviation; PSQI, Pittsburgh Sleep Quality Index; ISI, Insomnia Severity Index; STAI, State-Trait Anxiety Inventory; BDI, Beck Depression Inventory (cutoff >17); IRI, Interpersonal Reactivity Index. Bold fonts indicates significant p values*.

* *Kruskal-Wallis non-parametric test was used to compare males and females due to differences in sample size. Cohen D_z_ is reported as a measure of effect size*.

In our sample, 459 participants identified as females and 460 as women (99.7% overlap). One hundred and twelve participants identified as males and 105 as men (93.7% overlap). The Kruskal-Wallis non-parametric tests yielded the same statistically significant differences as the biological sex comparison. Please refer to Supplemental for complete statistics.

We also observed that straight participants reported the lowest quality of sleep (5.9 ± 2.7 vs. 7.1 ± 2.9 vs. 6.5 ± 2.7, respectively) and gay participants reported the highest insomnia symptoms (7.3 ± 4.8 vs. 9.4 ± 5.5 vs. 8.0 ± 4.4, respectively). The total score on the IRI was highest for bisexual/pansexual participants (63.1 ± 15.2 vs. 62.0 ± 15.0 vs. 71.8 ± 18.9, respectively). The sample size for these subgroups is however small and conclusions cannot be drawn.

### Changes in Sleep, Mood, and Empathy Over the Course of the Isolation Period

**Figure 1:**
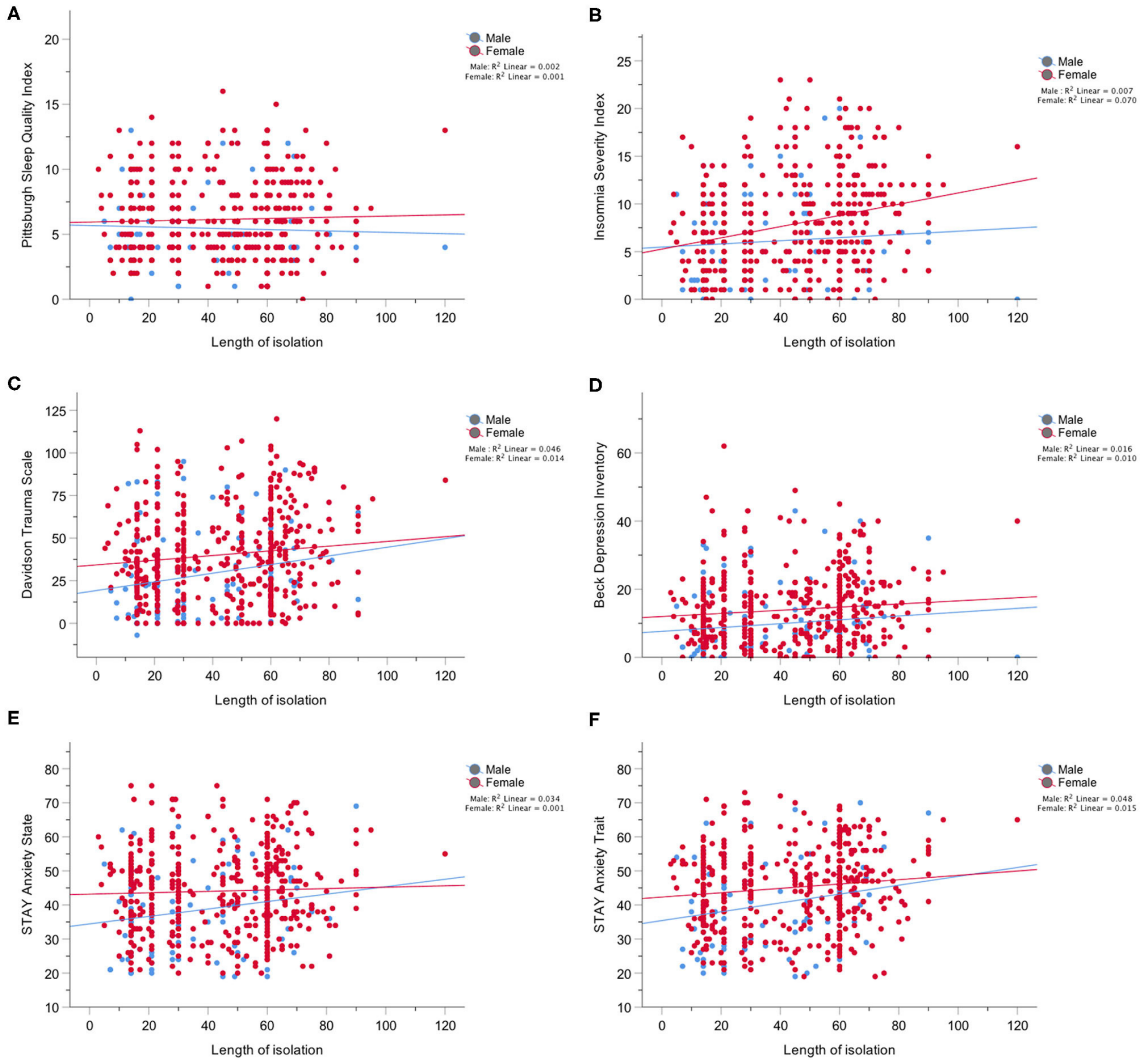
**(A)** Depicts the lack of changes over the course of the pandemic in the scores on the Pittsburgh Sleep Quality Index in males and females; **(B)** Depicts changes over the course of the pandemic in the scores on the Insomnia Severity Index which were significant only in females; **(C)** Depicts changes over the course of the pandemic in the scores on the Davidson Trauma Scale in both males and females; **(D)** Depicts changes over the course of the pandemic in the scores on the Beck Depression Inventory which were significant only in females; **(E)** Depicts lack of changes over the course of the pandemic in the scores on the STAI Anxiety State scale for both males and females; **(F)** Depicts changes over the course of the pandemic in the scores on the STAI Anxiety Trait scale for both males and females.

In male participants, we saw a worsening of trauma severity (β = 0.208, *p* = 0.033), trauma frequency (β = 0.209, *p* = 0.032), and trait anxiety (β = 0.220, *p* = 0.027) with increasing length of the isolation/social distancing period. In females, symptoms of insomnia (β = 0.264, *p* < 0.001), trauma severity (β = 0.136, *p* = 0.004) and frequency (β = 0.097, *p* = 0.041), symptoms of depression (β = 0.102, *p* = 0.029), and trait anxiety (β = 0.121, *p* = 0.011) progressed over the course of the isolation period. No changes with increased length of the isolation period were found in the IRI total score and subscales for both males and females (Figure 1).

### Predictors of Sleep Quality in the Whole sample

Please refer to Tables 4, 5 for complete statistics.

**Table 4:** Multiple Linear Regressions results in the whole sample.

**Whole sample**	**Age**	**Depression**	**Anxiety state**	**Anxiety trait**	**Trauma tot**	**IRI**	**Total model**
PSQI total	β = 0.167 ***p*** **<** **0.001** VIF = 1.022	β = 0.270 ***p*** **<** **0.001** VIF = 2.998	β = 0.119 ***p*** **=** **0.037** VIF = 2.045	β = 0.068 *p* = 0.334VIF = 3.859	β = 0.225 ***p*** **<** **0.001** VIF =2.045	β = −0.001 *p* = 0.968VIF = 1.117	*F*_1, 532_ = 4.367, ***p*** **=** **0.037**, *r* = 0.563, *r*^2^ = 0.317
Latency	β = −0.171 *p* = 0.104 VIF = 1.000	β = 0.037 *p* = 0.550VIF = 1.991	β = −0.022 *p* = 0.694 VIF = 1.680	β = −0.031 *p* = 0.585VIF = 1.728	β = 0.108 ***p*** **=** **0.013** VIF = 1.000	β = 0.191 *p* = −0.060VIF = 1.108	*F*_1, 524_ = 6.193, ***p*** **=** **0.013**, *r* = 0.130, *r*^2^ = 0.017
Duration	β = −0.292 ***p*** **<** **0.001** VIF = 1.014	β = −0.135 ***p*** **=** **0.032** VIF = 2.412	β = −0.148 ***p*** **=** **0.019** VIF = 2.412	β = 0.034 *p* = 0.669VIF = 3.842	β = 0.005 *p* = 0.932 VIF = 2.045	β = 0.017 *p* = 0.683VIF = 1.100	*F*_1, 533_ = 4.642, ***p*** **=** **0.032**, *r* = 0.371, *r*^2^ = 0.138
Efficiency	β = −0.129 ***p*** **=** **0.002** VIF = 1.012	β = −0.206 ***p*** **<** **0.001** VIF = 1.012	β = −0.082 *p* = 0.211 VIF = 2.412	β = −0.098 *p* = 0.167VIF = 2.842	β = 0.036 *p* = 0.542 VIF = 1.962	β = 0.007 *p* = 0.882 VIF = 1.099	*F*_1, 534_ = 23.653, ***p*** **<** **0.001**, *r* = 0.231, *r*^2^ = 0.053
ISI	β = 0.053 ***p*** **=** **0.118** VIF = 1.022	β = 0.282 ***p*** **<** **0.001** VIF = 2.995	β = 0.158 ***p*** **=** **0.001** VIF = 2.507	β = 0.088 *p* = 0.178VIF = 3.855	β = 0.275 ***p*** **<** **0.001** VIF = 2.045	β = 0.022 *p* = 0.538VIF = 1.117	*F*_1, 533_ = 8.991, ***p*** **=** **0.003**, *r* = 0.639, *r*^2^ = 0.409

*PSQI, Pittsburgh Sleep Quality Index; ISI, Insomnia Severity Index; VIF, variance inflation factor. Bold fonts indicates significant p values*.

**Table 5:** Multiple Linear Regressions results in Males and Females, separately.

**Males**	**Age**	**Depression**	**Anxiety state**	**Anxiety trait**	**Trauma tot**	**IRI**	
PSQI total	β = 0.026 *p* = 0.751 VIF = 1.003	β = 0.581 ***p*** **<** **0.001** VIF = 1.003	β = 0.183 *p* = 0.117 VIF = 1.989	β = 0.169 *p* = 0.206VIF = 2.611	β = 0.047 *p* = 0.730 VIF = 2.718	β = 0.069 *p* = 0.428VIF = 1.098	*F*_1, 97_ = 49.128, ***p*** **<** **0.001**, *r* = 0.580, *r*^2^ = 0.336
Latency	β = −0.206 ***p*** **=** **0.042** VIF = 1.000	β = 0.179 *p* = 0.074VIF = 1.003	β = 0.119 *p* = 0.237 VIF = 1.002	β = 0.084 *p* = 0.406VIF = 1.017	β = 0.137 *p* = 0.172 VIF = 1.001	β = 0.047 *p* = 0.649VIF = 1.055	*F*_1, 96_ = 4.243, ***p** **=*** **0.042**, *r* = 0.206, *r*^2^ = 0.042
Duration	β = −0.240 ***p*** **=** **0.016** VIF =1.000	β = 0.178 *p* = 0.070VIF =1.003	β = −0.176 *p* = 0.073 VIF =1.001	β = −0.158 *p* = 0.110VIF = 1.016	β = −0.098 *p* = 0.323 VIF =1.001	β = −0.061 *p* = 0.545VIF = 1.044	*F*_1, 98_ = 5.998, ***p*** **=** **0.016**, *r* = 0.240, *r*^2^ = 0.058
Efficiency	β = −0.207 ***p*** **=** **0.035** VIF = 1.016	β = −0.077 *p* = 0.620VIF = 2.577	β = −0.074 *p* = 0.714 VIF = 4.327	β = −0.279 ***p*** **=** **0.005** VIF =1.016	β = 0.094 *p* = 0.447 VIF = 1.082	β = −0.057 *p* = 0.573VIF =1.082	*F*_1, 97_ = 8.294, ***p*** **=** **0.005**, *r* = 0.326, *r*^2^ = 0.106
ISI	β = −0.091 *p* = 0.255 VIF = 1.003	β = 0.609 ***p*** **<** **0.001** VIF = 1.003	β = 0.148 *p* = 0.190 VIF = 1.989	β = −0.184 *p* = 0.154VIF = 2.611	β = −0.059 *p* = 0.656 VIF = 2.718	β = −0.060 *p* = 0.476VIF = 1.098	*F*_1, 97_ = 58.327, ***p*** **<** **0.001**, *r* = 0.621, *r*^2^ = 0.385
**Females**	**Age**	**Depression**	**Anxiety state**	**Anxiety trait**	**Trauma tot**	**IRI**	
PSQI total	β = 0.190 ***p*** **<** **0.001** VIF = 1.024	β = 0.315 ***p*** **<** **0.001** VIF =1.832	β = 0.102 *p* = 0.115 VIF =2.602	β = 0.103 *p* = 0.133VIF = 2.925	β = 0.278 ***p*** **<** **0.001** VIF =1.804	β = −0.010 *p* = 0.807VIF =1.096	*F*_1, 431_ = 26.758, ***p*** **<** **0.001**, *r* = 0.558, *r*^2^ = 0.312
Latency	β = −0.037 *p* = 0.446 VIF = 1.000	β = 0.019 *p* = 0.777VIF = 1.840	β = 0.042 *p* = 0.496 VIF = 1.648	β = −0.012 *p* = 0.844VIF = 1.696	β = 0.130 ***p*** **<** **0.007** VIF = 1.000	β = −0.034 *p* = 0.496VIF = 1.088	*F*_1, 424_ =7.303, ***p*** **=** **0.007**, *r* = 0.136, *r*^2^ = 0.018
Duration	β = −0.302 ***p*** **<** **0.001** VIF = 1.017	β = −0.142 ***p*** **=** **0.020** VIF = 2.478	β = −0.142 ***p*** **=** **0.044** VIF = 2.477	β = 0.007 *p* = 0.937VIF = 3.529	β = −0.028 *p* = 0.650 VIF = 1.895	β = 0.012 *p* = 0.791VIF = 1.080	*F*_1, 431_ = 4.581, ***p*** **=** **0.033**, *r* = 0.387, *r*^2^ = 0.150
Efficiency	β = −0.114 ***p*** **=** **0.017** VIF = 1.016	β = −0.181 ***p*** **<** **0.001** VIF = 1.016	β = −0.043 *p* = 0.561 VIF = 2.477	β = −0.055 *p* = 0.489VIF = 2.828	β = 0.012 *p* = 0.856 VIF = 1.804	β = 0.028 *p* = 0.572VIF = 1.080	*F*_1, 432_ =15.269, ***p*** **<** **0.001**, *r* = 0.205, *r*^2^ = 0.042
ISI	β = 0.078 **p** **=** **0.037** VIF = 1.028	β = 0.256 ***p*** **<** **0.001** VIF = 2.902	β = 0.147 ***p*** **=** **0.013** VIF = 2.592	β = 0.087 *p* = 0.208VIF =3.560	β = 0.328 ***p*** **<** **0.001** VIF = 1.895	β = 0.031 *p* = 0.419VIF = 1.097	*F*_1, 431_=6.184, ***p*** **=** **0.013**, *r* = 0.649, *r*^2^ = 0.421

*PSQI, Pittsburgh Sleep Quality Index; ISI, Insomnia Severity Index; VI, variance inflation factor. Bold fonts indicates significant p values*.

In the whole sample, after controlling for age, total PSQI scores were positively associated with depression symptoms, total trauma, and state anxiety (*p* = 0.037). Sleep latency was positively associated with total trauma (*p* = 0.013). Sleep duration was negatively associated with state anxiety and depression symptoms (*p* = 0.032). Sleep efficiency was negatively associated with depression symptoms (*p* < 0.001). Similarly, symptoms of insomnia were also positively associated with depression symptoms, total trauma, and state anxiety (*p* = 0.003).

### Predictors of Sleep Quality in the Two Sexes

In males, after controlling for age, total PSQI score, and insomnia symptoms were positively associated with depression symptoms (both *p* < 0.001). Sleep efficiency was instead negatively associated with trait anxiety (*p* = 0.005).

In females, total PSQI scores were positively associated with depression symptoms (*p* < 0.001). Sleep latency was associated with total trauma (*p* = 0.007). Sleep duration was negatively associated with depression symptoms and state anxiety (*p* = 0.033). Sleep efficiency was only negatively associated with depression symptoms (*p* < 0.001). Symptoms of insomnia were associated with total trauma, depression symptoms, and state anxiety (*p* = 0.013).

### Exploratory Correlational Analysis

We did not find associations between IRI empathy scores and the sleep variables in the whole sample. IRI scores had small positive correlations with PSQI total score (*r* = 0.086, *p* = 0.039), and symptoms of insomnia (*r* = 0.133, *p* = 0.001) indicating that worse quality of sleep was associated with greater IRI empathy scores. However, when the IRI scores were added to the regression models together with the other predictors they were not significantly associated with the sleep variables. IRI scores were in fact positively associated with trauma severity (*r* = 0.216*, p* < 0.001), and frequency (*r* = 0.269, *p* < 0.001), with depression scores (*r* = 0.316, *p* < 0.001) and state (*r* = 0.210, *p* < 0.001) and trait (*r* = 0.247, *p* < 0.001) anxiety. Among all subcomponents (see Supplementary Material), only *Personal Distress* was positively associated with sleep duration (*r* = 0.087, *p* = 0.038), sleep latency (*r* = 0.085, *p* = 0.044), total PSQI scores (*r* = 0.090, *p* = 0.032), and insomnia symptoms (*r* = 0.166, *p* < 0.001).

## Discussion

We examined sex and gender differences in the effects of the isolation period implemented in Canada to stop the spread of the COVID-19 on sleep, mood, and emotions. We found that compared to males, females reported lower quality of sleep, sleep efficiency, and greater symptoms of insomnia. They also reported significantly higher symptoms of anxiety, depression, and greater distress in relation to a traumatic event. In addition, females reported higher scores on the IRI empathy scale and all its subcomponents. Similar results were found when analyzing gender identity differences due to the great overlap between biological sex and gender identity in our sample. Over the course of the isolation period, sleep, and mood worsened, especially in females. Finally, we found that the most significant predictors of poor quality of sleep during the isolation were depression, anxiety and trauma scores. There were no statistically significant associations between IRI empathy scores and sleep variables, nor associations with symptoms of insomnia. A separate correlation analysis showed that higher IRI empathy scores were associated with higher depression anxiety and trauma scores.

To our knowledge, the sex and gender differences in sleep, mood, and emotions during the isolation in response to COVID-19 are novel findings, together with the assessment of the progression of sleep and mental health concerns with increasing days spent in isolation, especially in females. These results complement preliminary data from the recent KKF Coronavirus poll (49) reporting that women worry more about the health of their family compared to men (68 vs. 56%, respectively) and worry more about losing income due to a workplace closure or reduced hours (50 vs. 42%%, respectively). Women, compared to men, also worry more about risk of exposure to Coronavirus (39 vs. 31%, respectively). Women, compared to men reported that worry or stress related to COVID-19 has had a major negative impact on their mental health (16 vs. 11%, respectively). The greater worry and anxiety in women in relation to their role as caregiver clearly reflects differences in gender roles and norms. Unfortunately, in our study we did not collect information on childbearing, role of caregiver in the household, household income, and occupation. Future study should collect this information to better understand gender related differences in responses to the pandemic.

We found that the most significant predictors of sleep quality during the isolation, were depression, anxiety, and trauma in the whole sample, and in females. In male participants, only depression symptoms seemed to play a greater role. Contrary to our previous findings in non-pandemic times (31), here we did not find any significant associations between empathy scores, as measured by the IRI, and sleep variables when simultaneously adding mental health predictors in the models. It is possible that individuals respond to the pandemic with fear and anxiety for their own well-being and that those fight-or-flight responses cause a greater impact on individuals' sleep quality than empathy for others as compared to non-pandemic times. This is confirmed by the positive associations between *Personal Distress* and sleep disturbances. Differently from *Empathic Concern* which is a feeling associated with concerns for others and therefore altruistic, *Personal Distress* is a feeling of distress caused by the suffering of others and motivated by the selfish need of reducing the observer distress. The positive association with poor sleep quality and insomnia therefore indicates that what kept people awake was their own feeling of distress. On the other hand, we found that individuals with higher IRI empathy scores reported higher scores on the anxiety, depression and post-traumatic stress disorder scales. Analogous results were found in a study investigating the overlapping neural network between empathy and anxiety (50). Moreover, another study found that adults who had experienced trauma during childhood reported greater empathy, compassion, and prosocial behavior (51). While heightened anxiety and trauma appear to be a disadvantage for emotional well-being, it is reasonable to think that perhaps individuals who are more anxious about their self and others' well-being will also experience more empathy for others. A phenomenon known as “post-traumatic growth” describes heightened optimistic feelings, prosocial behavior, and trust for the humanity after traumatic events such as terroristic attacks (52–54).

The positive correlation that we found between anxiety and IRI empathy scores may also translate in greater following of the public health rules to protect oneself and individuals at higher risk. In a recent study by Harper et al. (55), the authors found that higher levels of anxiety and fear in response to the pandemic were the only predictors of positive behavior change including adherence to social distancing and greater hand washing practice. Similarly, Oosterhoff et al. (56) reported that the greatest motivators for adolescents in the United States to follow social distancing rules were prosocial motivations including social responsibility and not wanting others to get sick, being in a city/state of lockdown and parental rules. Adolescents that reported following the public health guidelines, reported greater anxiety when the motivation for isolation was fear of getting sick, but also reported feelings of belongingness to the community as a motivation for following public health guidelines. Future studies should directly test how heightened anxiety, empathy, and prosocial behavior are associated with social responsibility behavior during the COVID-19 pandemic. This direct analysis could inform about the importance of media messaging about empathy and caring for vulnerable population as a means to increase social distancing (57).

Our study has some limitations. While we focused specifically on the effects of the isolation on sleep and mental well-being, it is hard to fully distinguish these effects from anxiety or fear reactions to the spread of the virus. A greater number of females completed the survey as compared to males representing a selection bias due to the fact that women are more prone to respond to surveys (58). However, this led to different sample sizes for males and females and the need to use non-parametric statistics to compare the two groups. The study sample was composed of mainly young and well-educated individuals in the Canadian population and therefore the result cannot be generalized to other countries. As mentioned above, we did not collect information on family/household demands or domestic violence, pregnancy and postpartum conditions and other gender related factors that may have allowed a better characterization of the gender differences. Future studies should consider this limitation and collect these data. We did not use a standard questionnaire to evaluate gender but only inquired about biological sex, gender identity and sexual identity through questions in the demographic questionnaire. The use of a standardized questionnaire may have led to different results. Moreover, this study is cross-sectional therefore the causal role of anxiety, depression and trauma on sleep quality cannot be examined. Most importantly, we do not have information about sleep quality, depression anxiety and trauma before the pandemic; is therefore hard to distinguish the effects of the pandemic from individuals' own characteristics. A better characterization of the mental health state before the pandemic would have led to a better insight on the actual changes with the isolation. We only measured subjective sleep quality with questionnaires; the use of objective measures of sleep could result in different associations. Finally, we used the IRI questionnaire to assess empathy, however this is not the best methodology due to the dynamic nature of emotions.

In summary, our study highlights sex and gender differences in sleep, mood, and emotions in response to the isolation period implemented in Canada to stop the spread of COVID-19, with females and women suffering from more of the negative impacts which increased with greater length of the isolation. Moreover, our data provide evidence that the greatest predictors of changes in sleep quality during the isolation period are heightened anxiety, depression, and trauma symptoms, especially in females. Higher anxiety, depression, and trauma were however positively associated with empathy, perhaps indicating a positive role of fear, and anxiety responses to a crisis.

## Data Availability Statement

The raw data supporting the conclusions of this article will be made available by the authors, without undue reservation.

## Ethics Statement

The studies involving human participants were reviewed and approved by Conjoint Faculties Research Ethics Board (REB20-0650). The patients/participants provided their written informed consent to participate in this study.

## Author Contributions

VG, AU, and GI designed the study. VG and AU dealt with data collection. VG analyzed and interpreted the data and drafted the manuscript. All authors reviewed the manuscript.

## Conflict of Interest

The authors declare that the research was conducted in the absence of any commercial or financial relationships that could be construed as a potential conflict of interest.

## References

[1] World Health Organization. Protocol for Assessment of Potential Risk Factors for Coronavirus Disease 2019 (COVID-19) Among Health Workers in a Health Care Setting, 23 March 2020. World Health Organization (2020).

[2] Cucinotta D Vanelli M. WHO declares COVID-19 a pandemic. Acta Bio-Medica: Atenei Parmensis. (2020) 91:157–60. 10.23750/abm.v91i1.9397 32191675 PMC7569573

[3] Lewnard JA Lo NC. Scientific and ethical basis for social-distancing interventions against COVID-19. Lancet Infect Dis. (2020) 20:631–3. 10.1016/S1473-3099(20)30190-0 32213329 PMC7118670

[4] Rubin GJ Wessely S. The psychological effects of quarantining a city. BMJ. (2020) 368:m313. 10.1136/bmj.m313 31992552

[5] Galea S Merchant RM Lurie N. The mental health consequences of COVID-19 and physical distancing: the need for prevention and early intervention. JAMA Int Med. (2020) 180:817–8. 10.1001/jamainternmed.2020.1562 32275292

[6] Altena E Baglioni C Espie CA Ellis J Gavriloff D Holzinger B. Dealing with sleep problems during home confinement due to the COVID-19 outbreak: practical recommendations from a task force of the European CBT-I academy. J Sleep Res. (2020) 29:e13052. 10.1111/jsr.13052 32246787

[7] Tempesta D Curcio G De Gennaro L Ferrara M. Long-term impact of earthquakes on sleep quality. PLoS ONE. (2013) 8:e55936. 10.1371/journal.pone.0055936 23418478 PMC3572187

[8] Matsumoto S Yamaoka K Inoue M Muto S Teikyo Ishinomaki Research G Teikyo Ishinomaki Research Group and Health and Life Revival Council in the Ishinomaki district (RCI). Social ties may play a critical role in mitigating sleep difficulties in disaster-affected communities: a cross-sectional study in the Ishinomaki area, Japan. Sleep. (2014) 37:137–45. 10.5665/sleep.3324 24470703 PMC3902873

[9] Belleville G Ouellet M-C Morin CM. Post-traumatic stress among evacuees from the 2016 Fort McMurray wildfires: exploration of psychological and sleep symptoms three months after the evacuation. Int J Environ Res Public Health. (2019) 16:1604. 10.3390/ijerph16091604 31071909 PMC6540600

[10] Casagrande M Favieri F Tambelli R Forte G. The enemy who sealed the world: Effects quarantine due to the COVID-19 on sleep quality, anxiety, and psychological distress in the Italian population. Sleep Med. (2020) 75:12–20. 10.2139/ssrn.3576805 32853913 PMC7215153

[11] Mazza C Ricci E Biondi S Colasanti M Ferracuti S Napoli C. A nationwide survey of psychological distress among italian people during the COVID-19 pandemic: immediate psychological responses and associated factors. Int J Environ Res Public Health. (2020) 17:3165. 10.3390/ijerph17093165 32370116 PMC7246819

[12] Rossi R Socci V Pacitti F Di Lorenzo G Di Marco A Siracusano A. Mental health outcomes among frontline and second-line health care workers during the coronavirus disease 2019 (COVID-19) pandemic in Italy. JAMA Network Open. (2020) 3:e2010185. 10.1001/jamanetworkopen.2020.10185 32463467 PMC7256664

[13] Rossi R Socci V Talevi D Mensi S Niolu C Pacitti F. COVID-19 pandemic and lockdown measures impact on mental health among the general population in Italy. Front Psychiatry. (2020) 11:790. 10.3389/fpsyt.2020.00790 32848952 PMC7426501

[14] Lai J Ma S Wang Y Cai Z Hu J Wei N. Factors associated with mental health outcomes among health care workers exposed to coronavirus disease 2019. JAMA Network Open. (2020) 3:e203976. 10.1001/jamanetworkopen.2020.3976 32202646 PMC7090843

[15] Liu N Zhang F Wei C Jia Y Shang Z Sun L. Prevalence and predictors of PTSS during COVID-19 outbreak in China hardest-hit areas: gender differences matter. Psychiatry Res. (2020) 287:112921. 10.1016/j.psychres.2020.112921 32240896 PMC7102622

[16] Wang C Pan R Wan X Tan Y Xu L Ho CS. Immediate psychological responses and associated factors during the initial stage of the 2019 coronavirus disease (COVID-19) epidemic among the general population in China. Int J Environ Res Public Health. (2020) 17:1729. 10.3390/ijerph17051729 32155789 PMC7084952

[17] Xiao H Zhang Y Kong D Li S Yang N. The effects of social support on sleep quality of medical staff treating patients with coronavirus disease 2019 (COVID-19) in January and February 2020 in China. Med Sci Monitor. 2020) 26:e923549-1–8. 10.12659/MSM.923921 32132521 PMC7075079

[18] Wenham C Smith J Morgan R. COVID-19: the gendered impacts of the outbreak. Lancet. (2020) 395:846–8. 10.1016/S0140-6736(20)30526-2 32151325 PMC7124625

[19] Norris CM Yip CY Nerenberg KA Clavel MA Pacheco C Foulds HJ. State of the science in women's cardiovascular disease: a Canadian perspective on the influence of sex and gender. J Am Heart Assoc. (2020) 9:e015634. 10.1161/JAHA.119.015634 32063119 PMC7070224

[20] Tannenbaum C Norris CM McMurtry MS. Sex-specific considerations in guidelines generation and application. Can J Cardiol. (2019) 35:598–605. 10.1016/j.cjca.2018.11.011 30910247

[21] Cai H. Sex difference and smoking predisposition in patients with COVID-19. Lancet Respir Med. (2020) 8:e20. 10.1016/S2213-2600(20)30117-X 32171067 PMC7103991

[22] Gargaglioni LH Marques DA. Let's talk about sex in the context of COVID-19. J Appl Physiol. (2020) 128:1533–8. 10.1152/japplphysiol.00335.2020 32437244 PMC7303729

[23] Shamay-Tsoory SG. The neural bases for empathy. Neuroscientist. (2011) 17:18–24. 10.1177/1073858410379268 21071616

[24] Baron-Cohen S. Essential Difference: Male and Female Brains and the Truth about Autism. New York, NY: Basic Books (2004).

[25] Smith A. Cognitive empathy and emotional empathy in human behavior and evolution. Psychol Rec. (2006) 56:3. 10.1007/BF03395534

[26] Dziobek I Rogers K Fleck S Bahnemann M Heekeren HR Wolf OT. Dissociation of cognitive and emotional empathy in adults with Asperger syndrome using the Multifaceted Empathy Test (MET). J Autism Dev Dis. (2008) 38:464–73. 10.1007/s10803-007-0486-x 17990089

[27] Shamay-Tsoory SG Aharon-Peretz J Perry D. Two systems for empathy: a double dissociation between emotional and cognitive empathy in inferior frontal gyrus versus ventromedial prefrontal lesions. Brain. (2009) 132:617–27. 10.1093/brain/awn279 18971202

[28] Goel N Rao H Durmer JS Dinges DF. Neurocognitive consequences of sleep deprivation. Semin Neurol. (2009) 29:320–39. 10.1055/s-0029-1237117 19742409 PMC3564638

[29] Simon EB Vallat R Barnes CM Walker MP. Sleep loss and the socio-emotional brain. Trends Cognit Sci. (2020) 24:435–50. 10.1016/j.tics.2020.02.003 32299657

[30] Guadagni V Burles F Ferrara M Iaria G. The effects of sleep deprivation on emotional empathy. J Sleep Res. (2014) 23:657–63. 10.1111/jsr.12192 25117004

[31] Guadagni V Burles F Valera S Hardwicke Brown E Campbell T Ferrara M. The relationship between quality of sleep and emotional empathy. J Psychophysiol. (2016) 31:158–166. 10.1027/0269-8803/a00017730118565

[32] Tamm S Nilsonne G Schwarz J Lamm C Kecklund G Petrovic P. The effect of sleep restriction on empathy for pain: An fMRI study in younger and older adults. Sci Rep. (2017) 7:12236. 10.1038/s41598-017-12098-9 28947790 PMC5612991

[33] Guadagni V Burles F Ferrara M Iaria G. Sleep quality and its association with the insular cortex in emotional empathy. Eur J Neurosci. (2018) 48:2288–300. 10.1111/ejn.14124 30118565

[34] Christov-Moore L Simpson EA Coudé G Grigaityte K Iacoboni M Ferrari PF. Empathy: gender effects in brain and behavior. Neurosci Biobehav Rev. (2014) 46(Pt 4):604–27. 10.1016/j.neubiorev.2014.09.001 25236781 PMC5110041

[35] Mong JA Cusmano DM. Sex differences in sleep: impact of biological sex and sex steroids. Philos Trans R Soc Lond B Biol Sci. (2016) 371:20150110. 10.1098/rstb.2015.0110 26833831 PMC4785896

[36] Zhang B Wing YK. Sex differences in insomnia: a meta-analysis. Sleep. (2006) 29:85–93. 10.1093/sleep/29.1.85 16453985

[37] Lozo T Komnenov D Badr MS Mateika JH. Sex differences in sleep disordered breathing in adults. Respir Physiol Neurobiol. (2017) 245:65–75. 10.1016/j.resp.2016.11.001 27836648

[38] Hajali V Andersen ML Negah SS Sheibani V. Sex differences in sleep and sleep loss-induced cognitive deficits: the influence of gonadal hormones. Horm Behav. (2019) 108:50–61. 10.1016/j.yhbeh.2018.12.013 30597139

[39] Rainville JR Hodes GE. Inflaming sex differences in mood disorders. Neuropsychopharmacology. (2019) 44:184–99. 10.1038/s41386-018-0124-7 29955150 PMC6235877

[40] Buysse DJ Reynolds CF III Monk TH Berman SR Kupfer DJ. The Pittsburgh Sleep Quality Index: a new instrument for psychiatric practice and research. Psychiatry Res. (1989) 28:193–213. 10.1016/0165-1781(89)90047-4 2748771

[41] Morin CM Belleville G Bélanger L Ivers H. The Insomnia Severity Index: psychometric indicators to detect insomnia cases and evaluate treatment response. Sleep. (2011) 34:601. 10.1093/sleep/34.5.601 21532953 PMC3079939

[42] Spira AP Beaudreau SA Stone KL Kezirian EJ Lui L-Y Redline S. Reliability and validity of the Pittsburgh Sleep Quality Index and the Epworth Sleepiness Scale in older men. J Gerontol A Biol Sci Med Sci. (2012) 67:433–9. 10.1093/gerona/glr172 21934125 PMC3309871

[43] Spielberger CD Gorsuch RL Lushene RE. Manual for the State-Trait Anxiety Inventory. Mountain View, CA: Consulting Psychologists Press Inc. (1970).

[44] Julian LJ. Measures of anxiety: State-Trait Anxiety Inventory (STAI), Beck Anxiety Inventory (BAI), and Hospital Anxiety and Depression Scale-Anxiety (HADS-A). Arthr Care Res. (2011) 63(Suppl. 11):S467–72. 10.1002/acr.20561 22588767 PMC3879951

[45] Beck AT Steer RA Carbin MG. Psychometric properties of the Beck Depression Inventory: Twenty-five years of evaluation. Clin Psychol Rev. (1988) 8:77–100. 10.1016/0272-7358(88)90050-5

[46] Davidson JR Tharwani HM Connor KM. Davidson Trauma Scale (DTS): normative scores in the general population and effect sizes in placebo-controlled SSRI trials. Depress Anxiety. (2002) 15:75–8. 10.1002/da.10021 11891997

[47] Kevan B. Consistency and factorial invariance of the Davidson trauma scale in heterogeneous populations: results from the 2010 Chilean earthquake. Int J Methods Psychiatr Res. (2017) 26:e1516. 10.1002/mpr.1516 27453581 PMC5637937

[48] Davis MH. A multidimensional approach to individual differences in empathy. JSAS Catalog Select Documents Psychol. (1980) 10:85.

[49] Hamel L Lopes L Muñana C Kates J Michaud J Brodie M. KFF Coronavirus Poll: March 2020 (2020).

[50] Knight LK Stoica T Fogleman ND Depue BE. Convergent neural correlates of empathy and anxiety during socioemotional processing. Front Hum Neurosci. (2019) 13:94. 10.3389/fnhum.2019.00094 30949039 PMC6438321

[51] Greenberg DM Baron-Cohen S Rosenberg N Fonagy P Rentfrow PJ. Elevated empathy in adults following childhood trauma. PLoS ONE. (2018) 13:e0203886. 10.1371/journal.pone.0203886 30281628 PMC6169872

[52] Fredrickson BL Tugade MM Waugh CE Larkin GR. What good are positive emotions in crises? A prospective study of resilience and emotions following the terrorist attacks on the United States on September 11th, 2001. J Personal Soc Psychol. (2003) 84:365–76. 10.1037/0022-3514.84.2.365 12585810 PMC2755263

[53] Páez D Basabe N Ubillos S González-Castro JL. Social sharing, participation in demonstrations, emotional climate, and coping with collective violence after the March 11th Madrid bombings 1. J Soc Issues. (2007) 63:323–37. 10.1111/j.1540-4560.2007.00511.x

[54] Vázquez C Pérez-Sales P Hervás G. Positive effects of terrorism and posttraumatic growth: an individual and community perspective. In: Joseph S, Linle PA, editors. Trauma, Recovery, and Growth: Positive Psychological Perspectives on Posttraumatic Stress. John Wiley & Sons Inc. (2008). p. 63–91. 10.1002/9781118269718.ch4

[55] Harper CA Satchell LP Fido D Latzman RD. Functional fear predicts public health compliance in the COVID-19 pandemic. Int J Ment Health Addict. (2020). 10.31234/osf.io/jkfu3. [Epub ahead of print]. 32346359 PMC7185265

[56] Oosterhoff B Palmer CA Wilson J Shook N. Adolescents' motivations to engage in social distancing during the COVID-19 pandemic: associations with mental and social health. J Adolesc Health. (2020) 67:179–85. 10.1016/j.jadohealth.2020.05.004 32487491 PMC7205689

[57] Zaki J. Catastrophe compassion: understanding and extending prosociality under crisis. Trends Cogn Sci. (2020) 24:587–9. 10.1016/j.tics.2020.05.006 32410822 PMC7221394

[58] Smith G. Does Gender Influence Online Survey Participation? A Record-Linkage Analysis of University Faculty Online Survey Response Behavior. ERIC Document Reproduction Service No. ED 501717 (2008).

