# Supplementary material for: Sleep Quality, Empathy, and Mood During the Isolation Period of the COVID-19 Pandemic in the Canadian Population: Females and Women Suffered the Most
Source: Front Glob Womens Health. 2020 Oct 23;1:585938. doi: 10.3389/fgwh.2020.585938 (PMC8593942; doi:10.3389/fgwh.2020.585938)
Supplement: Supplementary file 1 [file Table_1.DOCX]

1. ***IRI subcomponents, mood and sleep correlations***

The *Perspective Taking* subscale was negatively associated with trait anxiety (*r* = -0.114, *p* = 0.008). The *Fantasy* subscale was positively associated with total PSQI scores (*r* = 0.121, *p* = 0.004) and insomnia symptoms (*r* = 0.158, *p* < 0.001). Higher *Fantasy* was also associated with higher trauma frequency (*r* = 0.241, *p* < 0.001), depression (*r* = 0.302, *p* < 0.001) and state (*r* = 0.179, *p* < 0.001) and trait anxiety (*r* = 0.266, *p* < 0.001). The *Empathic Concern* subscale was positively associated with trauma severity (*r* = 0.116, *p* = 0.006), frequency (*r* = 0.145, *p* = 0.001), and depression (*r* = 0.172, *p* < 0.001). Finally, *Personal Distress* was positively associated with sleep duration (*r* = 0.087, *p* = 0.038), sleep latency (*r* = 0.085, *p* = 0.044), total PSQI scores (*r* = 0.090, *p* = 0.032) and insomnia symptoms (*r* = 0.166, *p* < 0.001). Higher *Personal Distress* was also associated with higher trauma severity (*r* = 0.209, *p* < 0.001) and frequency (*r* = 0.273, *p* < 0.001), depression (*r* = 0.299, *p* < 0.001) and state (*r* = 0.367, *p* < 0.001) and trait anxiety (*r* = 0.406, *p* < 0.001). In summary, empathy in general, and all its subscales, were positively associated with increased mood disturbances. The only associations with sleep outcomes was a positive association between personal distress and poor sleep quality and symptoms of insomnia.

1. ***Gender identity differences***

In our sample, 459 participants identified as females and 460 as women. One hundred and twelve participants identified as males and 105 as men. The Kruskal-Wallis non-parametric tests yielded the same statistically significant differences as the biological sex comparison. See Table below.


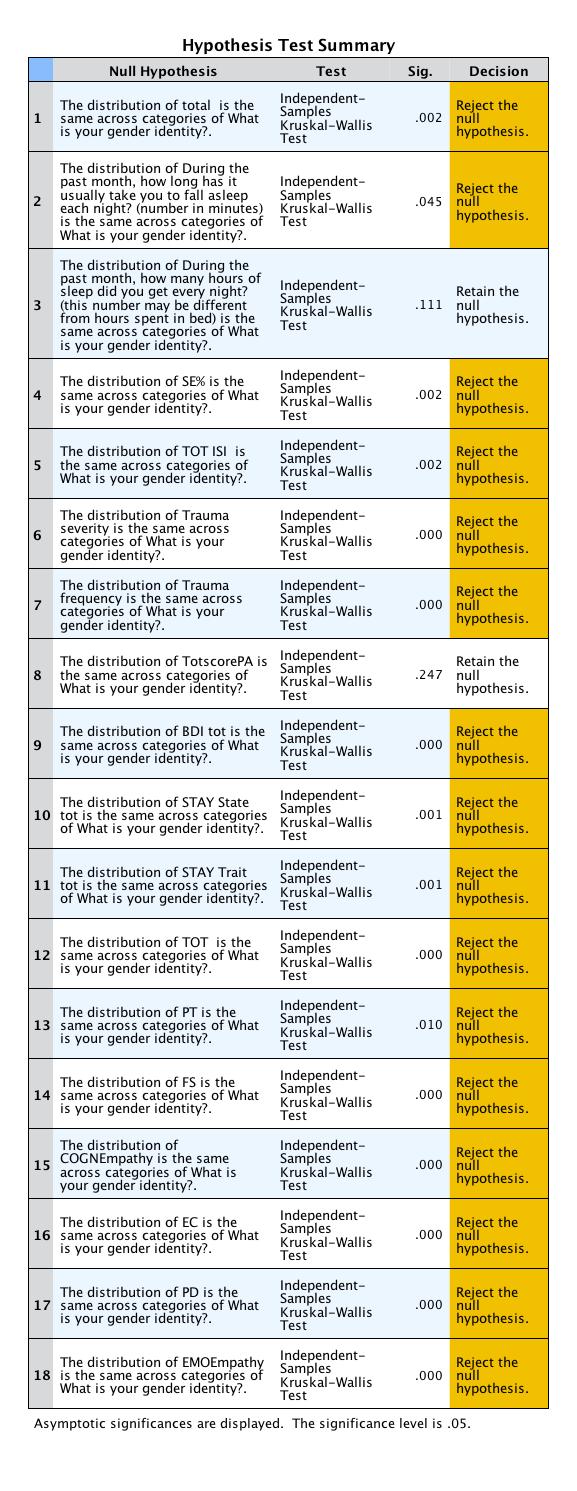
**es**
